# Protective Role of Hydrogen Sulfide against Noise-Induced Cochlear Damage: A Chronic Intracochlear Infusion Model

**DOI:** 10.1371/journal.pone.0026728

**Published:** 2011-10-26

**Authors:** Xu Li, Xiao-Bo Mao, Ren-Yi Hei, Zhi-Bin Zhang, Li-Ting Wen, Peng-Zhi Zhang, Jian-Hua Qiu, Li Qiao

**Affiliations:** 1 Department of Otolaryngology-Head and Neck Surgery, Xijing Hospital, Fourth Military Medical University, Xi'an, China; 2 Perimed China Ltd, Beijing, China; University of Southern California, United States of America

## Abstract

**Background:**

A reduction in cochlear blood flow plays an essential role in noise-induced hearing loss (NIHL). The timely regulation of cochlear perfusion determines the progression and prognosis of NIHL. Hydrogen sulfide (H_2_S) has attracted increasing interest as a vasodilator in cardiovascular systems. This study identified the role of H_2_S in cochlear blood flow regulation and noise protection.

**Methodology/Principal Findings:**

The gene and protein expression of the H_2_S synthetase cystathionine-γ-lyase (CSE) in the rat cochlea was examined using immunofluorescence and real-time PCR. Cochlear CSE mRNA levels varied according to the duration of noise exposure. A chronic intracochlear infusion model was built and artificial perilymph (AP), NaHS or DL-propargylglycine (PPG) were locally administered. Local sodium hydrosulfide (NaHS) significantly increased cochlear perfusion post-noise exposure. Cochlear morphological damage and hearing loss were alleviated in the NaHS group as measured by conventional auditory brainstem response (ABR), cochlear scanning electron microscope (SEM) and outer hair cell (OHC) count. The highest percentage of OHC loss occurred in the PPG group.

**Conclusions/Significance:**

Our results suggest that H_2_S plays an important role in the regulation of cochlear blood flow and the protection against noise. Further studies may identify a new preventive and therapeutic perspective on NIHL and other blood supply-related inner ear diseases.

## Introduction

Noise-induced hearing loss (NIHL) is a sensorineural hearing loss that results from noise-induced cochlear hair cell damage. An increasing number of individuals suffer from NIHL, which creates a great economic burden and a poor quality of life. Therefore, an investigation of the prevention and potential therapies of NIHL is warranted.

Temporary or permanent sensorineural hearing loss that is induced by exposure to noise depends on multiple factors, including noise parameters, living habits and genetic susceptibility [1], [2]. Although the exact pathological mechanism of NIHL is not known, direct mechanical trauma, metabolic stress and disorders of cochlear blood flow have been suggested [3]–[5]. Interestingly, each of these theories involves a disability of cochlear microvascular regulation, which may play an important role in NIHL. Vascular regulation includes vasoconstriction and vasodilation. Endothelin, α-adrenergic receptors, peptide-containing nerve fibers, and sphingosine-1-phosphate receptors participate in the constriction of spiral modiolar artery (SMA) [6]–[12], and nitric oxide (NO) and calcitonin gene-related protein (CGRP) regulate the relaxation of SMA [13], [14]. However, these factors do not exert a vasodilator effect on the SMA that is timely, rapid and strong enough to provide cochlear protection against noise.

Hydrogen sulfide (H_2_S) is a poisonous and occasionally lethal gas that is physiologically synthesized in blood vessels by cystathionine-γ-lyase (CSE) from L-cysteine. H_2_S activates ATP-sensitive potassium channel (K_ATP_) and transient receptor potential (TRP) channels to exert vasodilatory effects [15], which may be initiated by hypoxia [16]. CSE knockout mice display hypertension and diminished endothelium-dependent vasodilation [17]. The administration of DL-propargylglycine (PPG), which is an inhibitor of CSE, recovers arterial pressure and heart rate in rats [18]. These results suggest that H_2_S is a physiological vasodilator.

However, whether H_2_S assumes the same responsibility in the cochlea remains to be elucidated. This study explored the regulatory effect of H_2_S on cochlear blood flow and identified the potential protective role of H_2_S against NIHL. These results provide a new preventive and therapeutic perspective for blood supply-related inner ear diseases.

## Results

### CSE protein expression in cochlea

**Figure 1:**
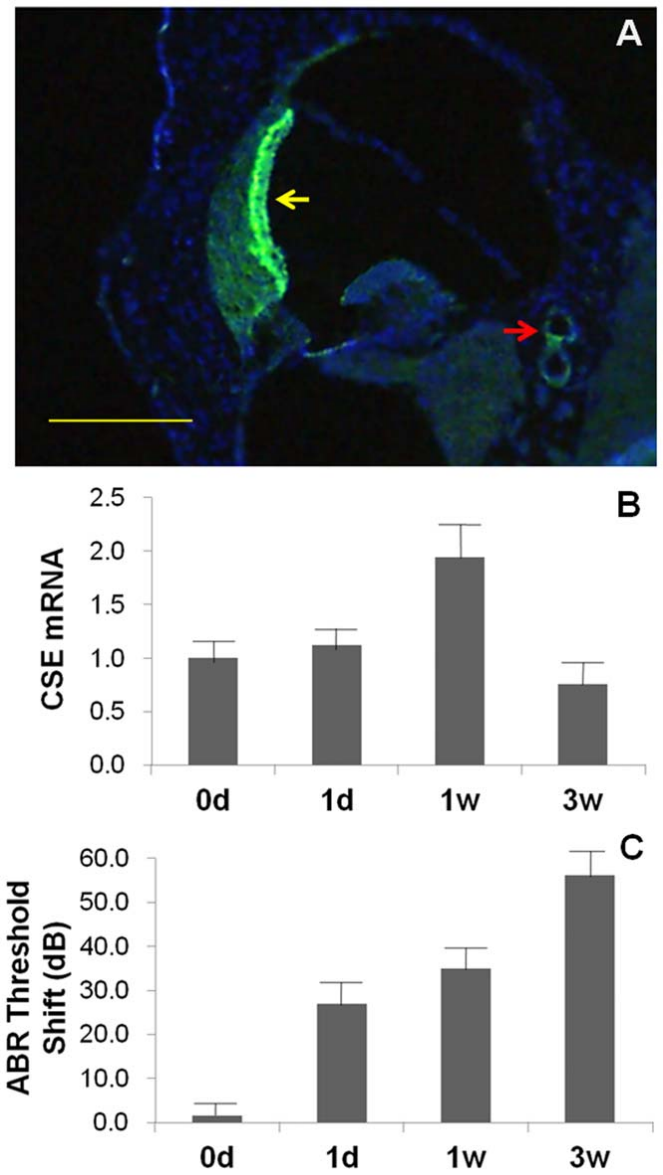
CSE expression in cochlea. A: Immunofluorescent photograph of CSE in the cochlea (bar = 200 µm). CSE protein was positively expressed in the stria vascularis (yellow arrow) and the wall of the spiral modiolar artery (red arrow). B and C are mean CSE mRNA expression in rat cochlea and ABR threshold shifts. The relative expressions after noise exposure for 0 d, 1 d, 1 w and 3 w were 1.00±0.17, 1.12±0.17, 1.95±0.31 and 0.76±0.19, respectively. ABR threshold shifts were 1.56±3.26, 26.88±5.79, 35.00±5.18 and 56.25±5.67 dB SPL, respectively. They both increased with increased exposure time initially. In the 3-week exposure group, the expression of CSE mRNA decreased inversely.

Immunofluorescence was used to examine the protein expression of CSE in cochlea. CSE protein was identified in the cochlear stria vascularis and the SMA wall (Fig. 1A). No immunofluorescence was observed in controls. The distribution of CSE protein corresponded with previous reports of CSE expression in the cardiovascular system [19].

### Cochlear CSE mRNA expression after different durations of noise exposure

To show that H_2_S may play a role in NIHL, cochlear CSE mRNA expression and auditory brainstem response (ABR) threshold shifts were analyzed after different durations of noise exposure. Cochlear CSE mRNA was assessed using real-time quantitative PCR, and the results are shown in Fig. 1B. CSE transcripts were detected in all samples. The relative expressions after noise exposure for 0 d, 1 d, 1 w and 3 w were 1.00±0.17, 1.12±0.17, 1.95±0.31 and 0.76±0.19, respectively. ABR threshold shifts were 1.56±3.26, 26.88±5.79, 35.00±5.18 and 56.25±5.67 dB SPL, respectively (Fig. 1C). CSE mRNA expression and ABR threshold shifts increased with the increased in exposure time when the noise stimulation lasted no more than 1 week. However, CSE mRNA expression decreased inversely in the 3-week exposure group. We ascribed this decrease to cochlear decompensation that resulted from noise overstimulation.

### H_2_S donor increased cochlear blood flow

**Figure 2:**
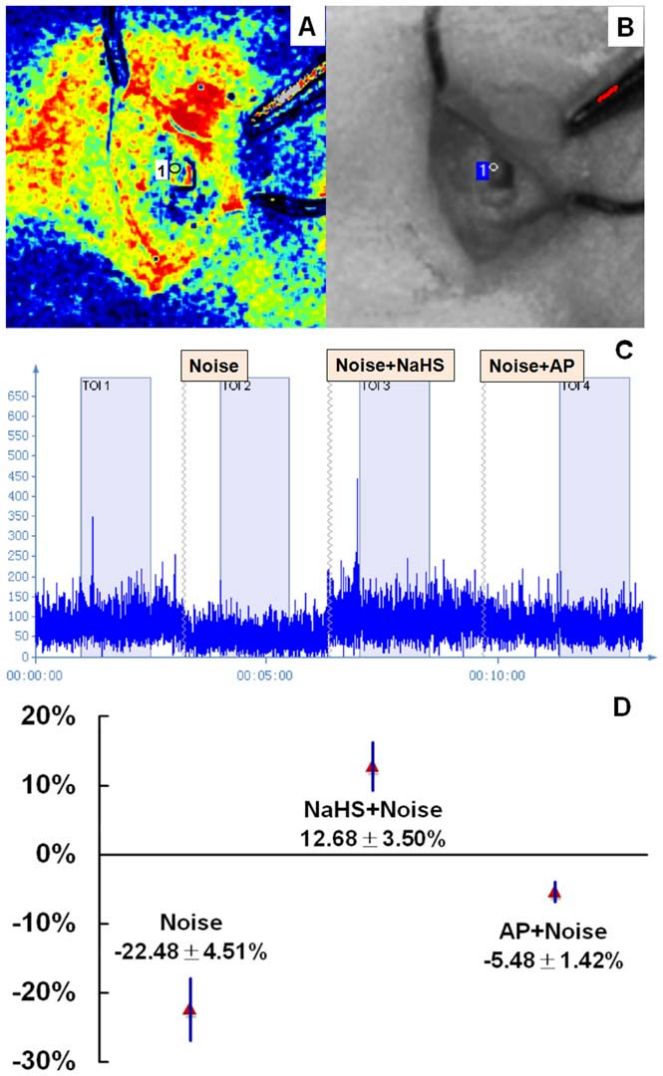
Detection of cochlear blood perfusion. The cochlear blood flow was detected before and after noise exposure. Post-exposure data were also recorded after previous NaHS or AP administration to identify the effect of H_2_S. A and B are a recorded blood perfusion image and color photograph, respectively. C represents cochlear blood flow in graphical form. X axis is time and Y axis is blood flow data. A blood flow reduction was observed immediately after noise exposure compared to the pre-exposure blood flow. D shows changes in post-exposure cochlear blood flow after different interventions. Cochlear post-exposure blood flow after NaHS administration increased significantly compared to that after AP administration (n = 7, P<0.05).

To reveal the effect of noise, the cochlear blood flow was detected in 7 cochleae before and after noise exposure. Post-exposure data were also recorded after previous sodium hydrosulfide (NaHS) or artificial perilymph (AP) administration to identify the effect of H_2_S. Blood perfusion image (Fig. 2A), color photograph (Fig. 2B) and blood flow (Fig. 2C) were presented respectively. An evident blood flow reduction was observed immediately after noise exposure compared to the pre-exposure blood flow. A progressive increase and gradual recovery was observed in the following 15–20 min. ANOVA indicated a significant difference in cochlear blood flow between control, AP, and NaHS administration (F = 26.79, p<0.001). Cochlear post-exposure blood flow after NaHS administration increased significantly compared to the administration of AP (SNK test, p<0.05) (Fig. 2D). The same result was observed using laser Doppler flowmetry (PeriFlux System 5000 and Probe 401; Perimed, Stockholm, Sweden).

### Morphological and functional protection of H_2_S against NIHL

A chronic intracochlear infusion model was built and AP, NaHS or DL-propargylglycine (PPG) was locally administered. To identify the morphological and functional protection of H_2_S against NIHL, ABR measurement, scanning electron microscope (SEM) and outer hair cell (OHC) count were carried out.

Two rats died of post-surgical infection, and one rat died of hemorrhage. The ABR threshold shifts immediately after surgery in the control^1^, NaHS^2^ and PPG^3^ groups were 7.78±1.88, 6.50±1.30 and 5.00±1.34 dB SPL, respectively. No significant differences between groups were observed (Equivalence test, 0.002<P_1, 2_<0.005, P_2, 3_<0.001, 0.002<P_1, 3_<0.005).

**Figure 3:**
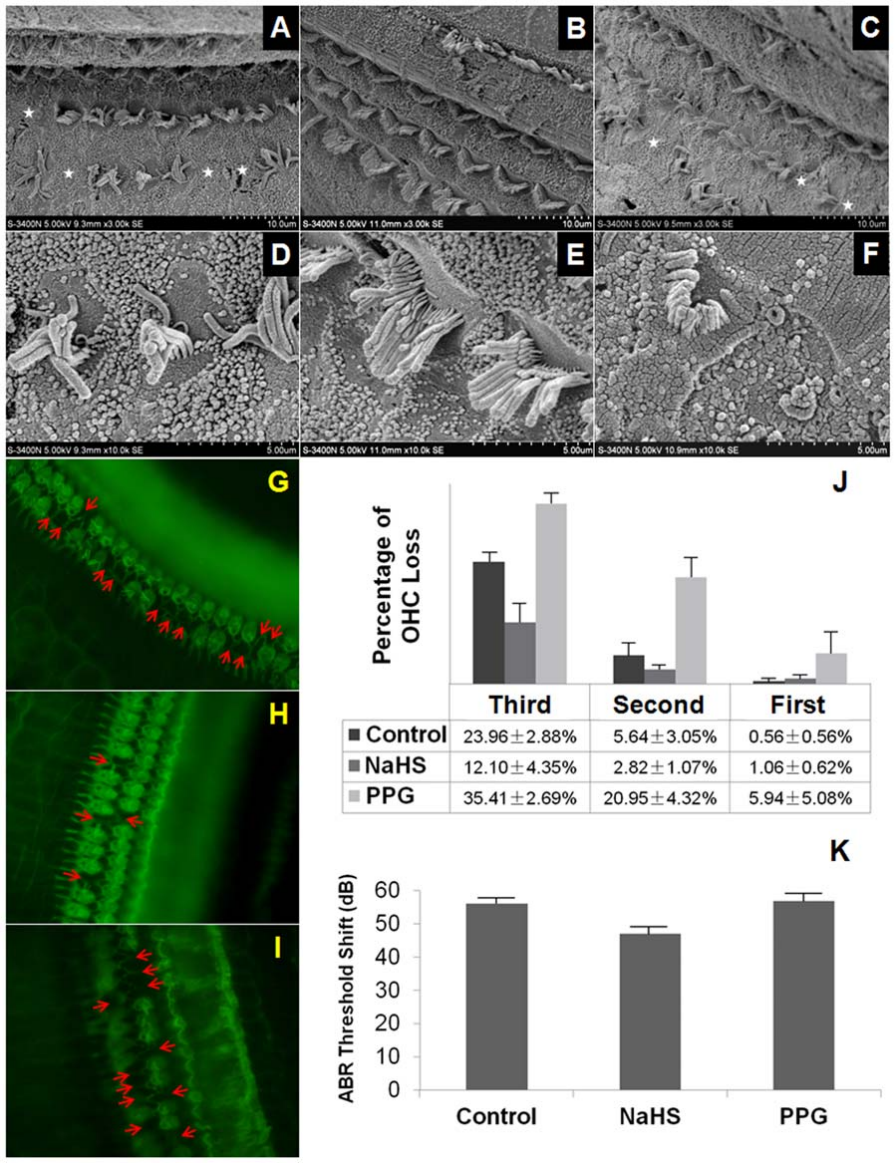
Morphological and functional protective effects of H_2_S against NIHL. A–F: Scanning electron micrographs of the basilar membrane. Evident stereocilia bundle defects (white stars) were observed in control (D, E) and PPG (H, I) groups. The stereocilia bundles in the NaHS group appeared relatively normal (F, G). G–I: Cochlear sensory epithelia surface preparation in control (A), NaHS (B) and PPG (C) groups. OHC loss and evident stereocilia bundle defects are indicated (red arrows). J: Percent OHC loss in each row. The loss was localized primarily in the third row and the posterior second row. OHC loss in the NaHS group is less than control. The PPG exhibited the highest loss percentage. K: Mean post-surgical ABR threshold shifts at different time points after noise exposure. The ABR threshold shift demonstrated a significant decrease on the 14th day in the NaHS group (47.00±2.60 dB SPL) compared to the control group (56.11±2.32 dB SPL) and the PPG group (56.88±2.82 dB SPL) (P<0.05).

SEM and cochlear surface preparation revealed missing hair cells primarily in OHCs but seldom in inner hair cells (IHCs) (Figs. 3A–C, G–I). The missing OHCs were localized primarily in the superior segment of the basal turn. In contrast to the evident stereocilia bundle defects in the control (Figs. 3D and G) or PPG (Figs. 3F and I) groups, the stereocilia bundles appeared reasonably normal in the NaHS group (Figs. 3E and H). The percent OHC loss in each row is listed in Fig. 3J. OHC loss was localized in the third row and the posterior second row, which demonstrated a different susceptibility of the OHCs in each row to noise. Statistical analyses revealed that the percent of OHC loss in the first row increased in the PPG group and decreased significantly in the NaHS group (ANOVA, F = 11.83, p<0.01; SNK test, p<0.05). The disarrangement of OHC stereociliary bundles was least obvious in the NaHS group compared to the other groups. These results suggested that exogenous H_2_S administration protected the cochlea against noise damage.

The ABR threshold shifts of post-surgical rats were recorded at different times after noise exposure (Fig. 3K). The auditory system recovered gradually. ANOVA indicated a significant difference in the ABR shift on the 14th day between the three groups (F = 4.71, p<0.05). There was no statistically significant difference between the control group (56.11±2.32 dB SPL) and the PPG group (56.88±2.82 dB SPL) (SNK, P>0.05). However, the ABR threshold shift demonstrated a significant decrease in the NaHS group (47.00±2.60 dB SPL) compared to the other groups (SNK, P<0.05). These results suggested a functionally protective effect of H_2_S against NIHL.

## Discussion

This study explored the potential action of H_2_S in the cochlea. The results demonstrated that the CSE protein was expressed abundantly in rat cochlea. CSE mRNA expression correlated with the duration of noise stimulation, which suggested its probable association with acoustic trauma. Exogenous H_2_S increased cochlear blood flow rapidly, which may relieve noise-induced blood flow decrease. The intracochlear administration of an H_2_S donor ameliorated noise-induced cochlear morphological damage and hearing impairment. Therefore, H_2_S may morphologically and functionally protect the cochlea against noise-induced damage. We conclude that the protection may result from its effect of cochlear microcirculation improvement.

H_2_S is a well-known environmental hazard and toxin. However, its essential physiological role as an endogenous gaseous transmitter has garnered much attention. H_2_S induces either relaxation or contraction depending on different concentrations and vessel type [20]–[23]. H_2_S regulates angiogenesis under physiological and ischemic conditions [24], and it may play an important role in atherosclerosis [25]. H_2_S demonstrates cytoprotective effect in various organ systems in ischemia/reperfusion injury [26]. Furthermore, H_2_S may exert a different action in various inflammatory states [27]–[29].

CSE activity determines H_2_S production in cardiovascular system. The timely, rapid and strong vasodilator effect of H_2_S likely satisfies the needs of cochlear blood flow autoregulation. The CSE inhibitor, PPG, regulates the endogenous production of H_2_S [30], [31]. In this study, cochlear protection was mainly induced by exogenous H_2_S, but the action of PPG was not obvious. We hypothesize that the greater loss of OHCs resulted from the direct action of PPG.

A laser speckle contrast blood perfusion imager was used to detect cochlear blood flow in this study. In contrast to laser Doppler flowmetry, the laser speckle contrast blood perfusion imager visualizes tissue blood perfusion in real-time and combines dynamic responses with spatial resolution.

Our results revealed the expression of CSE in the cochlea and the role of H_2_S in the regulation of cochlear blood flow. Further studies may provide a new preventive and therapeutic perspective on NIHL and other blood supply-related inner ear diseases.

## Materials and Methods

### Animals and Ethics Statement

SD rats weighing 250–350 g were used in this study. All rats were provided by the animal center of the Fourth Military Medical University and had free access to water and food. All procedures concerning animals in this study were approved by the Institutional Animal Care and Use Committee of the Fourth Military Medical University (Permit number, SYXK 2008-005) in compliance with the Guide for the Care and Use of Laboratory Animals.

### Immunological studies

Rats were anesthetized with a 50 mg/kg intraperitoneal injection of sodium pentobarbital (catalog number P11011, Merck, Germany). Perfusions through the heart were performed with freshly prepared 4% phosphate-buffered paraformaldehyde (pH 7.4). The temporal bones were removed immediately and were post-fixed in the same solution overnight. After decalcification in 10% ethylene diamine tetraacetic acid (EDTA) for 7 d and dehydration in 30% sucrose for 24 h, immunofluorescence was performed on cochlear frozen sections (8 µm thickness). The sections were blocked with 0.3% peroxide in methanol and 10% normal goat serum successively. The sections were incubated overnight at 4°C with a mouse monoclonal anti-CSE antibody (catalog number H00001491-M01, Abnova, Taiwan, China). A fluorescein isothiocyanate (FITC)-conjugated goat anti-mouse IgG (Sigma Chemical Co., St. Louis, MO, USA) was used as a secondary antibody. A Hoechst (Sigma Chemical Co., St. Louis, MO, USA) was used to stain the nucleus. After several rinses in PBS, the sections were photographed using an Olympus BX51 compound microscope.

### Noise exposure

Rats were separately placed into wire mesh cages in a ventilated chamber with free access to food and water during noise exposure. A RadioShack Super-tweeter located above the cages generated a noise (4 kHz octave band, 120 dB SPL) for 6 h. The noise was amplified using a power amplifier (Yamaha AX-500U) and delivered to a loudspeaker. The homogeneity of the sound field was confirmed using a sound-level meter (Bruel and Kjaer Type 2606) that was placed at various locations within the chamber.

### ABR measurements

After an intraperitoneal injection of sodium pentobarbital (40 mg/kg), the reference, ground and active needle electrodes were inserted beneath the skin of the post-measured auricle, the sacrococcygeal region and the calvaria, respectively. The responses to 1024 click presentations were amplified, filtered, and synchronously averaged. Each stimulus was presented initially at 100 dB SPL. The stimulus intensity was decreased systematically in 5-dB steps until a visually discernible ABR waveform disappeared. ‘Threshold’ was defined as the lowest level of the stimulus that produced a visually detectable response.

### RNA isolation and real-time quantitative PCR

Total RNA was isolated from the stria vascularis and basilar membrane using TRIZOL reagent (Takara Shuzo, Shiga, Japan). Reverse transcription was performed using a PrimeScript RT Master Mix (Takara Shuzo, Shiga, Japan) according to the manufacturer's instructions. Total RNA (1 µg) was reverse transcribed into cDNA in each sample. Controls that contained no reverse transcriptase were used to safeguard for genomic DNA contamination. Real-time PCR was performed in an iCycler iQ Real-time PCR Detection (Bio-Rad, Hercules, CA) associated with the iCycler optical system software (version 3.1) using SYBR Premix Ex Taq II according to the protocol. Briefly, all PCRs were performed in a volume of 25 µl. The cycling was conducted at 95°C for 90 s followed by 38 cycles of 95°C for 10 s and at 60°C for 20 s. The primers of CSE were as follows: sense 5′-AGCGATCACACCACA-GACCAAG-3′ and antisense 5′-ATCAGCACCCAGAGCCAAAGG-3′. These primers produced a product of 178 bp. The primers for rat β-actin were sense 5′-CGTTGACATCCGTAAAGAC-3′and antisense 5′-TAGGAGCCAGGGCAGTA-3′. Specificity of the amplification was determined using a melting curve analysis. Data are expressed as a ratio of the quantity of CSE mRNA to the quantity of β-actin mRNA using the arithmetic formula 2^−ΔΔ*CT*^.

### Detection of cochlear blood flow

After deep anesthesia and post-auricular skin preparation, an incision was performed to expose the temporal bone. The bulla was opened to reveal the round window and the basal turn of the cochlea. The cochlear mucoperiosteum was uncovered under a microscope. Cochlear blood flow was recorded before and after noise exposure (120 dB SPL, 4 kHz for 10 min) using a laser speckle contrast blood perfusion imager (PeriCam PSI; Perimed, Stockholm, Sweden). Signal amplitudes that backscattered from the cochlea were calculated using the manufacturer's software (PimSoft 1.2.2.5676; Perimed, Stockholm, Sweden). A piece of gel foam (3 mm×3 mm) that was immersed in 0.3 ml AP or NaHS (1 mmol/L) was placed on the round window. Blood flow was detected again after the same noise stimulation was applied. Twenty-five min between neighboring noise exposures were reserved for the recovery of cochlear blood flow.

### Surgery and experimental group

Thirty rats were divided randomly into 3 groups (control, NaHS and PPG group). The chronic intracochlear infusion models were designed according to the method described by Prieskorn et al. [32]. Briefly, the right post-auricular region was shaved and cleaned for surgical manipulation after deep anesthesia. Lidocaine hydrochloride (1%) was injected subcutaneously to provide local anesthesia. A post-auricular incision was performed, and the muscles were separated to expose the temporal bone. The bulla was perforated using a 1 mm diamond paste burr for visualization of the round window and the cochlear basal turn. A small fenestra was created using a sharpened probe on the basal turn of the scala tympani approximately 1.0 mm below the round window. The tip of a mouse jugular catheter (Order No.: 0007700, Alza Corp., CA, USA) was inserted into the scala tympani and connected to a mini-osmotic pump #2002 (Alza Corp., CA, USA), which was embedded hypodermically. The pumps and catheters were filled with 200 µl AP, NaHS (1 mol/L) or PPG (1 mol/L) 12 h before implantation and primed according to the manufacturer's recommendations. Sterile working conditions were maintained throughout the entire procedure. Animals were exposed to noise (120 dB SPL, 4 kHz, 6 h) the following day. The ABR thresholds were recorded pre-surgery and post-surgery on the 1st, 3rd, 5th, and 14th d after noise exposure. OHC counts and cochlear SEM were performed after the last ABR measurement.

### Cochlear sensory epithelia surface preparation and OHC count

The animals were perfused transcardially with freshly prepared 4% phosphate-buffered paraformaldehyde (pH 7.4) under deep anesthesia. The right cochleae were removed immediately. Cochleae were perfused locally through the open round windows and cochlear apexes to ensure efficient fixative perfusion and post-fixed in the same stationary liquid overnight. After removal of the bony capsule, the spiral ligament, stria vascularis and Reissner's membrane were separated under a dissecting microscope. Each turn of the Corti organ was detached from the bony modiolus. The sensory epithelium was trimmed, and surface preparations were stained for actin using fluoresceinyl-aminomethyldithiolano-phalloidin (catalog number Alx-350-268-MC01, Enzo Life Sciences, Farmingdale, NY, USA). The sensory epithelia surface structures were carefully examined for missing cells or stereocilia under a fluorescence microscope (Olympus, Tokyo, Japan) at a magnification of 400×. The missing hair cells and stereocilia were quantified and photographed along the entire basilar membrane. The percent missing OHCs in each row were calculated and compared between the three groups.

### SEM study

Deeply anesthetized animals were decapitated on the 15th day after noise exposure. The right cochleae were removed immediately and gently perfused with 2.5% phosphate-buffered glutaraldehyde (catalog number A17876, Alfa-Aesar, USA) (pH 7.4) through the open round window and the cochlear apex. The cochleae remained in the same solution overnight. The bony capsule was removed after washing with 0.1 M phosphate-buffered saline (PBS). The spiral ligament and stria vascularis were removed under a dissecting microscope. The Reissner's membrane was separated. The dissected specimens were rinsed with 0.1 M PBS, post-fixed in 1% osmium tetroxide for 2 h and placed in 2% tannic acid twice for 30 min. The cochleae were dehydrated in a series of graded ethanol solutions and dried in a critical point drier (hcp-2, Hitachi). The specimens were fixed on a metal stage, gold-coated in a sputter coater (E102 Ion Sputter, Hitachi) and observed under a SEM (Hitachi S-800).

### Statistical analyses

Data are presented as the mean ± SE. The equivalence test assessed the interclass differences of surgical impact on the auditory system. ANOVA followed by the SNK test identified the differences between means. A value of *P*<0.05 was considered statistically significant. The analyses were performed using a commercial statistical software package (SPSS 13.0; SPSS Inc., Chicago, Ill.).
